# On-body measure of reaction time correlates with intoxication level

**DOI:** 10.1371/journal.pone.0323858

**Published:** 2026-04-15

**Authors:** Megan H. Blackwell, Henry L. Valk, Joshua Eaton, Samuel J. Kovaly, Samuel J. Karnes, Tue Vu, Kate M. Stevenson, Michael P. Kowalczyk, William B. Gormley

**Affiliations:** 1 Pison Technology, Inc., Boston, Massachusetts, United States of America; 2 Department of Neurosurgery, Brigham and Women’s Hospital, Boston, Massachusetts, United States of America; Universidad Autonoma de Chihuahua, MEXICO

## Abstract

Excessive alcohol use has profound effects on individual health and healthcare systems worldwide. Despite this, there is currently no system or device that can detect robustly the physiologic and functional effects of alcohol-based impairment in real-world conditions. A practical, on-body, device capable of rapidly and accurately determining the functional capacity of an individual to drive, before they can start the ignition of the automobile, is required. The goal of this pilot study was to evaluate the effect of acute alcohol intoxication on premotor time (PMT) and reaction time (RT), both highly sensitive of individual cognition, as evaluated by the Pison Technology wrist-worn wearable. Nineteen participants (9 male, 10 female) between the ages of 21 and 36 years were recruited for this study, including 14 subjects who consumed alcohol sufficient to raise blood alcohol concentration (BAC) to 0.10-0.12% within a 30-minute period and 5 controls who did not consume alcohol. Changes in reaction time data were correlated with blood alcohol levels as measured by breathalyzer testing, identifying a statistically significant difference between those participants under the legal limit and those over the legal limit (F(3, 3545) = 117, p < 0.001). The results indicated a significant effect of BAC level on reaction time change (Delta RT), Post hoc Tukey tests showed that participants in the medium (M = 104.4; 95% CI, 99.2--109.6) and high (M = 112.7; 95% CI, 107.5--117.9) BAC groups responded significantly slower than controls (M = 72.6; 95% CI, 69.9--75.3; p < 0.05 for both comparisons). The low BAC group (M = 67.5; 95% CI, 65.1--69.9) also differed from controls (p < 0.05). No significant difference was observed between the medium and high BAC groups (p = 0.13). Overall, reaction times increased as BAC levels rose. Both group and individual analyses confirmed that as the BAC increased in subjects, the PMT also increased. The PMT also decreased as the BAC returned to levels under the legal intoxication threshold (0.08%). The effect of BAC on PMT was significant, F(1, 2161) = 156, p < 0.001, with a partial η² of 0.067, indicating a moderate effect size, and a corresponding Cohen’s *d* of approximately 0.52, indicating a medium-sized difference between under-limit and over-limit conditions. This study demonstrated the first use case of an on-body, neuro-physiological sensor capable of detecting sensitive changes of reaction time, in real time, that serves as an easy-to-measure proxy for blood alcohol content and impairment.

## Introduction

According to the Center for Disease Control, excessive alcohol use resulted in over 140,000 deaths and 3.6 million years of potential life lost each year in the United States from 2015 to 2019, and over 178,000 deaths and 4.3 million years of potential life lost in the United States from 2020-2021, with younger populations bearing a disproportionate portion of this risk [1]. Worldwide, 13.5% of total deaths in the 20–39 age group were attributed to alcohol use [2,3]. In 2020 alone, there were 11,654 fatalities in traffic crashes in which at least one driver was alcohol-impaired, with a blood alcohol concentration (BAC) exceeding 0.08 g/dL [4]. This seemingly preventable loss of life motivated the 2021 Infrastructure Investment and Jobs Act to require inclusion in all new passenger cars a technology that can “passively monitor the performance of a driver … to accurately identify whether that driver may be impaired” and “prevent or limit motor vehicle operation if an impairment is detected” [5]. To achieve the vision of eliminating drunk driving requires the development of technologies that are easy to use and provide user-specific data in a timely fashion. Despite the rapid advancement and proliferation of sensor technology, there is currently no system or device that unobtrusively and inconspicuously assesses impairment created by alcohol consumption to 1) enable users to refrain from consuming more alcohol when impairment is detected and 2) give individuals quantitative data on their ability (or lack thereof) to manage equipment, operate machinery, and drive.

Although two emerging technologies were identified by the National Highway Traffic Safety Administration and the Driver Alcohol Detection System for Safety as promising candidates for ignition interlock devices, each presents challenges in the robust detection of intoxication [6]. TruTouch, a technology that uses diffuse reflectance spectroscopy at the finger, suffers from weak ethanol absorption and confounding absorptions from other skin tissue components, such as melanin, and hydration [7]. Measurements from Autoliv, a technology that uses non-dispersive infrared gas sensors on ambient air, have a large variability due to challenges in sample collection and complex calibration [8]. Dilution of the driver’s breath is also a problem that decreases Autoliv’s sensitivity. These passive devices do not require active participation by drivers, but each presents detection challenges. These systems must have a high sensitivity to detect driver alcohol impairment while also reducing the false-positive rate of detection to be used as an effective ignition interlock device. Consequently, there remains an unmet need for an easy-to-use yet robust sensor to detect alcohol impairment.

A direct and more reliable way to measure impairment associated with increased blood alcohol levels is to measure reaction time rather than measuring alcohol itself. Reaction time is crucial in preventing motor vehicle accidents [9,10]. Studies have investigated driver reaction time to avoid a collision and measured a mean time of 2.2 seconds required to apply brakes and 1.6 seconds to steer away [11]. Several studies have measured the relationship between blood alcohol concentration (BAC) and reaction time, linking increasing alcohol intake with slower stimulus-response behavior [10,12–14]. A BAC of 0.06% increased brake reaction time by 20% [15]. Reaction time alterations are widely understood to be altered early and reliably in cases of alcohol intoxication.

Pison has recently developed a wearable device that can detect increases in reaction time, which accompany any intoxication, specifically ones caused by alcohol. Intoxication assessment can be accomplished by a patient-initiated reaction time test and the results compared to a baseline value. One envisioned end use of the Pison wearable device provides users with direct information to promote healthy choices concerning alcohol use and increase public safety. Another end use advances the device as an alcohol or fatigue interlock for motor vehicles. Pison aims to commercialize the first ignition-interlock device that infers impairment from increased reaction time measurements acquired on its wrist-worn sensor. Another envisioned use for this technology is by transportation organizations that wish to assess drivers or operators for readiness to increase the safety of operations.

This study aims to quantify the accuracy of reaction time-based models to predict blood alcohol concentration in real time. The study is a comparison group study designed to collect laboratory, physiologic, and psychometric subject data during progressive administration of weight- and gender-based oral ethanol to an experimental arm and compare performance against controls who were not provided alcohol. Subjects in the experimental arm were also used as their own control to measure the progressive effects of alcohol on reaction time. In this pilot study, physiologic and behavioral biomarkers collected using the Pison device investigate the relationship between blood alcohol levels as measured by a breathalyzer and physiological changes as measured by electroencephalography (EEG). Shifts in alpha band activity have been reported in the early stages of intoxication and in the theta band after BACs of 0.08% and higher are reached [16]. The time course of alcohol-induced changes as measured by BAC and EEG will be compared with changes in measured reaction time. Results from the EEG study will be reported in a separate paper. The EEG markers may be used to train machine learning models to guide the interpretation of the reaction-time changes for improved accuracy in identifying intoxication.

**Fig 1:**
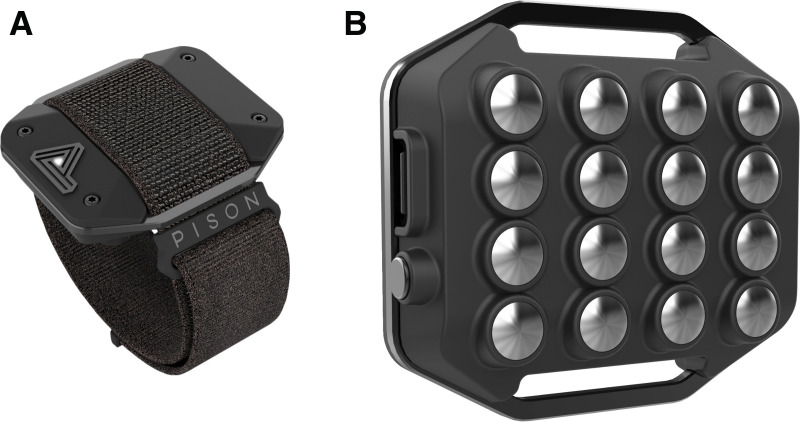
Pison wearable device. Pison’s sensor is worn on the wrist and uses stainless steel electrodes to measure biopotential activity on the top of the wrist. Reprinted with permission from Pison Technology, original copyright 2021.

The Pison wearable collects multi-channel surface electromyography (EMG) measurements from the peripheral nervous system. The Pison wearable used in this study (Fig 1) includes five channels of biosignal data measured at 1 kHz sampling rate from differential pairs of electrodes on the surface of the skin. Filtering and preprocessing of sensor data are performed on device, resulting in high-quality EMG signals. The device also contains a 9-axis inertial measurement sensor that samples at 90 Hz. No calibration is required. The Pison wearable is paired to a compatible mobile phone to which sensor readings are shared via Bluetooth.

**Fig 2:**
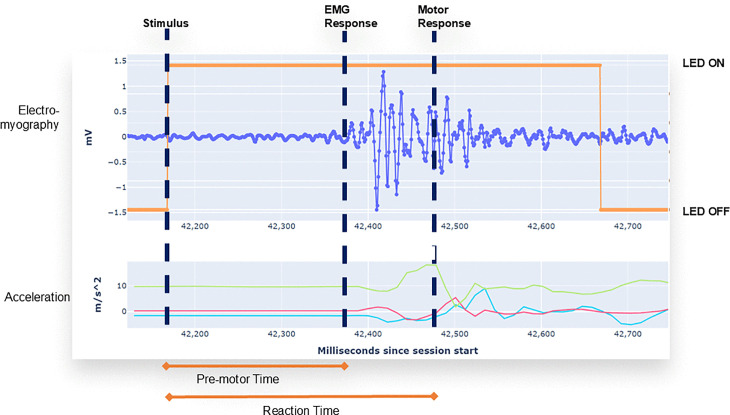
Illustration of premotor and reaction times. The Pison wearable uses surface electromyography (EMG) and accelerometry data to measure premotor time (PMT) in response to a visual stimulus presented on the device. Premotor time describes the time elapsed between the visual stimulus and the neuromuscular response as measured by an increase in the EMG signal. Response onset is determined by Pison’s proprietary algorithm. Reaction time is determined by measuring the onset of movement as measured by the accelerometer signal. Electromechanical delay describes the difference between the pre-motor time and reaction time.

The Pison wearable uses EMG and accelerometry data to measure premotor time (PMT) and reaction time (RT) in response to a visual stimulus presented on the device (Fig 2). Premotor time describes the time elapsed between the visual stimulus and the neuromuscular response as measured by an increase in the EMG signal. Reaction time is determined by measuring the onset of movement as measured by the accelerometer signal. Electromechanical delay is defined as the time between EMG onset and the onset of force or motion and is estimated by the difference between the measured premotor and reaction times [17]. The Pison wearable offers reaction time tests to users as a fast, 30-second “Readiness” test or a longer 3-minute “Focus” test. A user’s consistency, false starts, and lapses are reported to estimate performance. The timing of the visual stimulus is randomized to have an inter-stimulus interval between 1 and 4 seconds.

The Pison reaction time assessment is entirely self-contained and does not rely on touchscreens or peripheral devices that can suffer from latencies due to operating system delays [18]. No calibration procedure is required. Pison’s device does not suffer from software or peripheral delays seen with current reaction-time tests that rely upon consumer-grade touchscreens, keyboards, or other inputs to measure a user’s response time. A potential source of error is electromagnetic interference. The Pison wearable is presently used as a personal wellness and training device but could be modified to support an ignition interlock device in future products.

**Fig 3:**
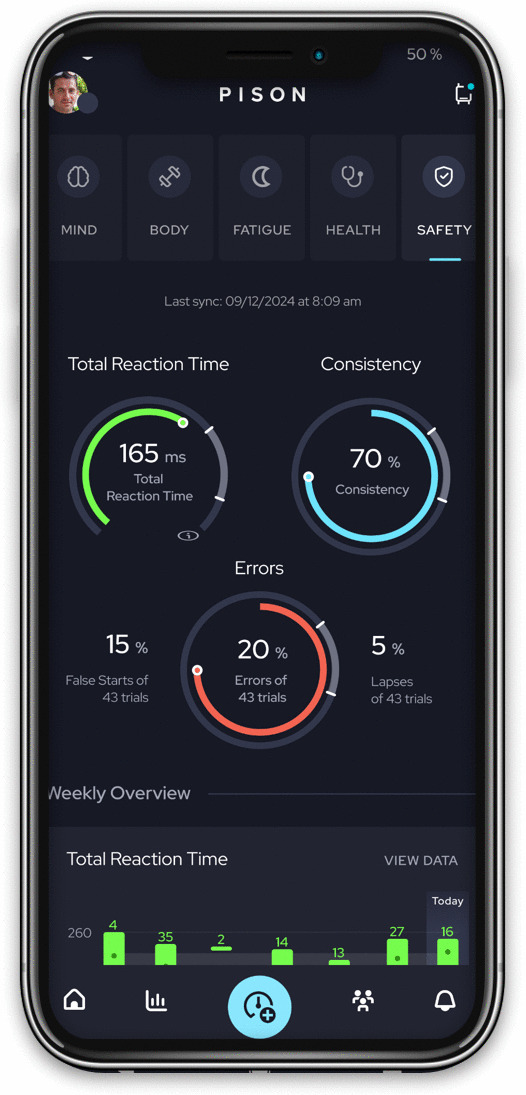
Pison mobile application. Pison’s mobile application quantifies readiness by comparing reaction-time measurements to a baseline data set. Reprinted with permission from Pison Technology, original copyright 2023.

A custom software application (Fig 3) running on a mobile device, such as a cell phone or tablet, displays premotor time and reaction time results as well as comparisons with baseline performance. A user may opt-in to share data with others who have proper permissions.

This study demonstrates the use of reaction time to measure impairment related to levels of blood alcohol exceeding the legal limit. The neurophysiologic data measured with the Pison wearable provides a reliable predictive capacity to determine, at a population level, in a statistically significant manner, the difference between subjects below and above the legal BAC. Additionally, at an individual level, a consistent mirroring of reaction time to blood alcohol level was shown.

## Materials and methods

### Participants

Ethical approval for this study was obtained from the Western-Copernicus Group Institutional Review Board (study number 1360480). Written informed consent was obtained from all participants. A total of 14 test subjects (6 males, 8 females) and five controls (3 males, 2 females) ranging in age from 21–36 years were recruited between September 26 and October 3, 2023; there were 8 males and 10 female participants who completed testing. Subjects were recruited through an agency and compensated $100 for study completion. Race or ethnicity was not collected. Those excluded from the study were students, active-duty military personnel, prisoners, cognitively impaired persons, women who were pregnant, and individuals who had backgrounds of alcohol or substance abuse. All subjects were screened for alcohol dependence through the Alcohol Dependence Scale during intake and excluded if scoring 8 or above. Women who consented to participate were provided a pregnancy test to be taken on-site. Subjects were asked to step on a scale to ascertain their body weight in pounds to support the calculation of a standardized and personalized alcohol dosage, as published [19]. The intent of the dosage was to produce a BAC over 0.10%, but ideally no greater than 0.12%, after 30 minutes from the time of consumption. One drink was equivalent to a 1.25 oz serving of an 80 Proof liquor, or a 12 oz serving of beer, or a 5 oz serving of wine. After each 40 min period of drinking, 0.01 gm% was subtracted from the estimated blood alcohol level.

Two days before the test, eight subjects who had never used the Pison wearable were familiarized with the device and reaction time tests. Baseline data were recorded. The remaining 11 subjects had previously collected baseline data. Subjects who were unable to retrieve a device prior to the day of intervention were asked to take baseline measurements two days after and have been noted in the data.

Subjects were instructed to complete an active RT test or “Salus Protocol” in the morning, afternoon, and evening leading up to the intervention to account for training effects on RT. The “Salus Protocol” includes passive EMG measurements of reaction time, recorded while participants positioned their arm in a “watch-check” position. During each active RT measurement, subjects were prompted by a 500 ms duration white LED light emitted by the wearable and asked to perform the specific action of opening their hand in response to the light as fast as possible. The active test lasted three minutes with a randomized inter-stimulus interval between 1 and 4 seconds.

### Protocol

**Fig 4:**
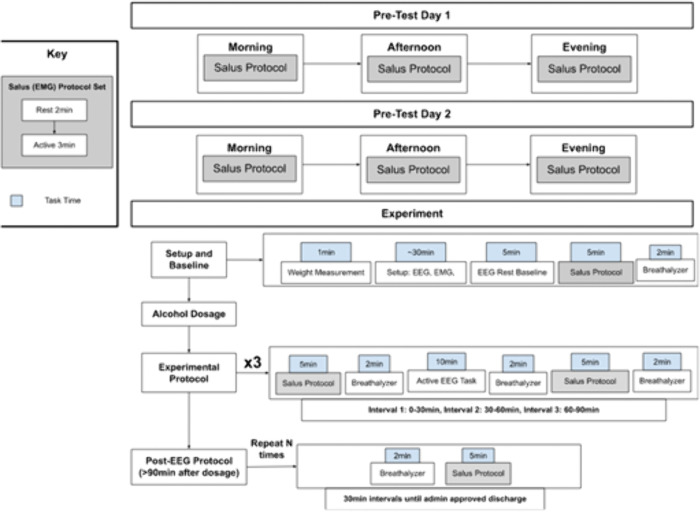
Experimental protocol of BAC study.

On the day of the experiment, intake questionnaires were completed and signed in private. All physiological data measures were labeled with the subject’s unique identification number, along with forms regarding the behavioral, physiological, and psychomotor data collected. Participants were instructed to not consume food 8 hours prior to their session in order to expedite the effects of alcohol. Alcohol was administered to participants but not to controls after obtaining baseline measurements for all tasks, in which 100% of the determined dose was consumed over the course of ten minutes. After dosage, a cyclic process of taking breathalyzer (BACTrack S80), EEG (32-channel Enobio, Neuroelectrics), and RT measurements was continued for the remainder of the experiment in alternating blocks performed every 15 minutes, as shown in Fig 4. In the first block, passive EMG, breathalyzer, and RT tests were administered. In the second block, an EEG task was also administered. This resulted in six passive, active, and breathalyzer measurements and three EEG measurements in the 90-minute session. Thirty minutes after the initial dosage, if the participants’ BAC had not reached 0.10% or greater, additional alcohol was administered based on a published standardized reference table, taking into account the participant’s current BAC, desired BAC, and body weight [19].

The BACTrack breathalyzer has a removable mouthpiece that was cleaned or replaced before each participant. The 32-channel Enobio EEG as used to capture brain activity. Lab Streaming Layer was used simultaneously with the headset’s software to synchronize and record the EEG measurements.

RT and PMT values were obtained from the Pison wearable recordings and compared against BAC levels measured by a breathalyzer. Statistical analyses were performed to evaluate the relationship between alcohol intoxication and changes in RT/PMT. Specifically, one-way and repeated-measures analysis of variance (ANOVA) tests were conducted to assess differences in RT across BAC categories (control, low, medium, high; and below vs. above legal threshold). Post hoc comparisons were performed using Tukey’s Honestly Significant Difference (HSD) test. Linear regression models were implemented to quantify the predictive capacity of BAC for changes in RT (Delta RT). Additionally, correlation analyses were conducted to evaluate the association between RT and PMT in control participants. Missing data points (e.g., failed sensor recordings) were excluded from analyses. Lastly, all statistical tests used a significance threshold of p < 0.05 and the models were developed using both R and Python.

## Results

### Reaction time and blood alcohol

**Fig 5:**
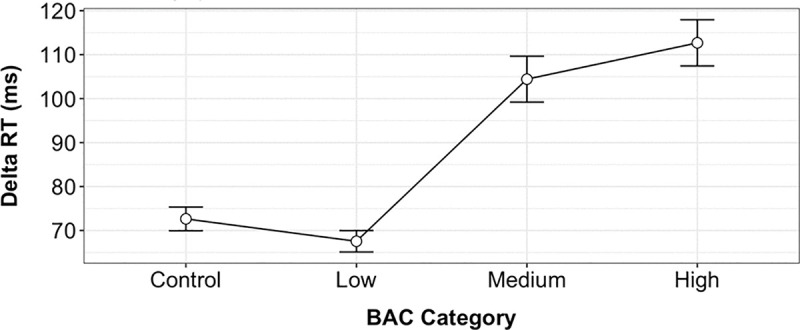
The effect of blood alcohol concentration level on reaction time. A one-way between-subjects analysis of variance (ANOVA) illustrating the effect of blood alcohol concentration (BAC) on changes in reaction time (RT) for low (BAC <= 0.05%), medium (0.05% < BAC <= 0.08%), and high (BAC > 0.08%) levels. Pairwise comparisons show statistically significant differences between each factor at the p < 0.05 level for the four conditions [F(3, 3545) = 117, p < 0.001].

All data collected for this study is freely accessible at http://www.pison.com/BAC-data. A one-way between-subjects analysis of variance (ANOVA) demonstrated a significant effect of BAC level on changes in reaction time, noted as “Delta RT,” F(3, 3545) = 117, p < 0.001 (Fig 5). Categories used include low (BAC <= 0.05%), medium (0.05% < BAC <= 0.08%), and high (BAC > 0.08%) levels. Changes in RT for the control group were also included in the comparison. Post hoc Tukey tests indicated that participants in the medium (M = 104.4; 95% CI, 99.2–109.6) and high (M = 112.7; 95% CI, 107.5–117.9) BAC groups responded significantly slower than controls (M = 72.6; 95% CI, 69.9–75.3; p < 0.05 for both comparisons). The low BAC group (M = 67.5; 95% CI, 65.1–69.9) also differed significantly from controls (p < 0.05). While Delta RT increased with BAC levels above 0.05%, no significant difference was observed between the medium and high BAC groups (p = 0.13).

**Fig 6:**
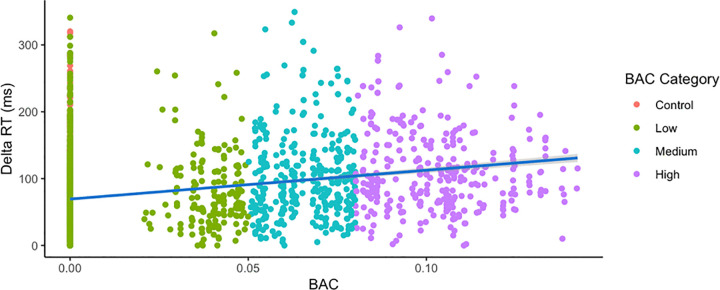
Blood alcohol content predicts changes in reaction time. Subject data was pooled for all participants and is plotted in red for control subjects, green for low levels (BAC <= 0.05%), teal for medium levels (0.05% < BAC <= 0.08%), and purple for high levels (BAC > 0.08%). A simple linear regression analysis was conducted to evaluate the extent to which BAC levels could predict the change in reaction time, denoted as “Delta RT.” A significant regression was found (F(1, 3547) = 323.3, p < 0.001), although only 8.4% of the variance was explained (R^2^ = 0.084).

A simple linear regression (Fig 6 showed that BAC level was a significant predictor of Delta RT, (F(1, 3547) = 323.3, p < 0.001), accounting for 8.4% of the variance (R^2^ = 0.084). The model intercept was 69.45 (95% CI, 67.60--71.29), representing the estimated baseline at zero BAC. For every 1% increase in BAC, Delta RT is predicted to increase by 431.65 units (95% CI, 384.58--478.71).

**Fig 7:**
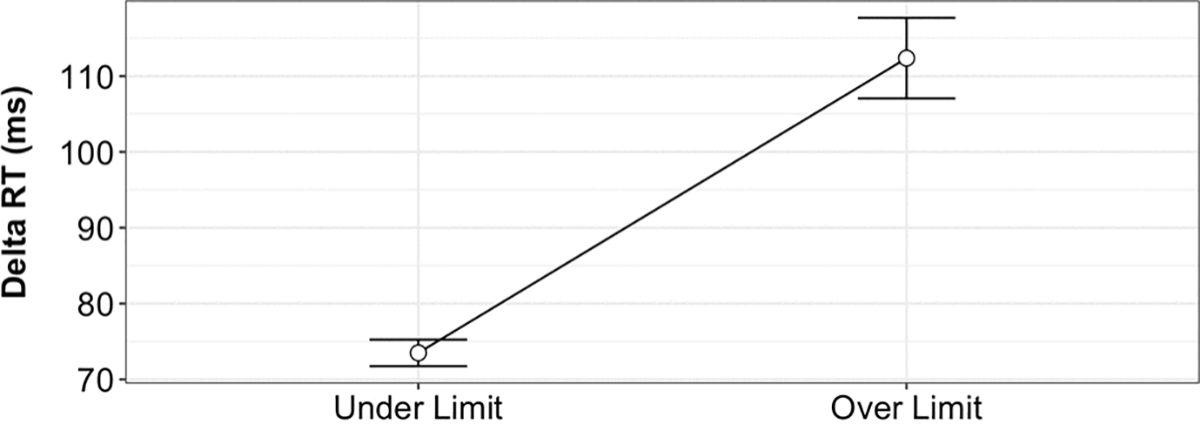
The separability of BAC level based on Delta RT. A one-way between-subjects ANOVA was conducted on the Delta RT for a group of participants while under the legal BAC limit (BAC <= 0.08%) and over the legal BAC limit (BAC ≥ 0.08%). Pairwise comparisons show statistically significant differences between each factor at the p < 0.05 level.

A one-way between-subjects ANOVA was conducted to compare the effect of BAC on Delta RT (Fig 7) for BAC values under the legal limit (BAC < 0.08%) and over the legal limit (BAC ≥ 0.08%). Participants exceeding the legal limit (BAC ≥ 0.08%, M = 112.4, SE = 2.71) showed significantly slower reaction times compared to those below the legal limit (M = 73.5, SE = 0.89), [F(1, 3547) = 185.3, p < 0.001]. The mean increase in Delta RT for those over the legal limit was 38.88 units (95% CI, 33.28–44.48) relative to the under-limit group (95% CI for mean, 71.71–75.24). This finding suggests that exceeding the legal BAC threshold is associated with a substantial slowing of reaction times.

**Fig 8:**
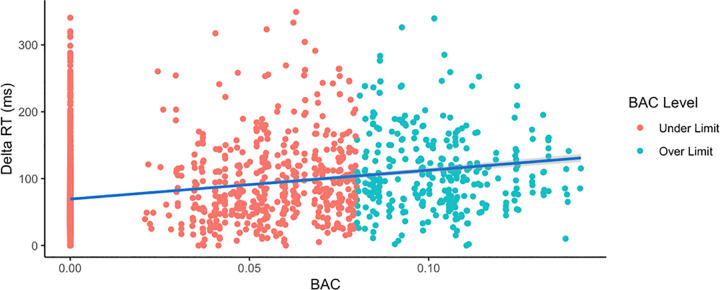
Linear regression analysis demonstrates the ability of blood alcohol content to predict changes in reaction time. Subject data was pooled for all participants, including controls, and is plotted in red for under the legal limit (BAC <= 0.08%) and in blue for over the legal limit (BAC > 0.08%) levels. A simple linear regression analysis was conducted to evaluate the extent to which BAC levels could predict the change in reaction time, denoted as “Delta RT.” A significant regression was found for the two conditions [F(1, 3547) = 323.3, p < 0.001], although only 8.3% of the variance was explained (R^2^ = 0.083).

A simple linear regression analysis was conducted to evaluate if BAC levels could predict the Delta RT between data from participants who were under or over the legal limit of 0.08% (Fig 8). Control participants are included in the analysis. A significant regression was found (*F*(1, 3547) = 323.3, *p* < 0.001), although only 8.3% of the variance was explained (R^2^ = 0.083).

### Premotor time and blood alcohol

**Fig 9:**
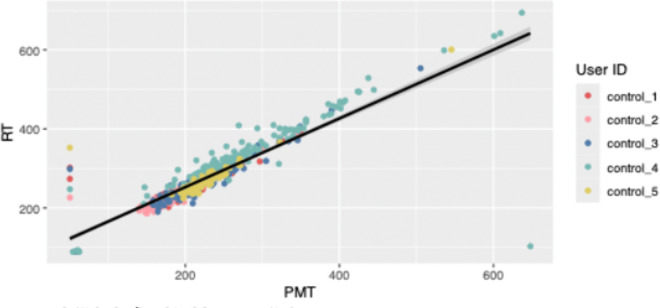
A correlation of 0.874 (p < 0.001) was established between premotor time and reaction time in control subjects, suggesting a consistent relationship and a consistent electromechanical delay.

**Fig 10:**
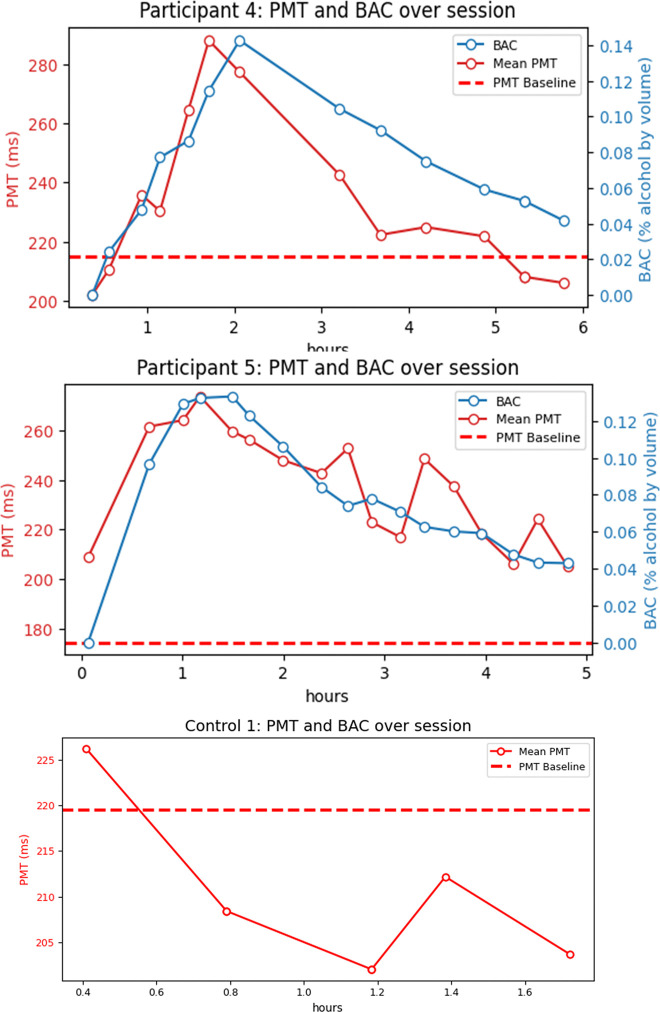
Blood alcohol concentration and reaction time plotted as a function of time. Blood alcohol concentration and reaction time were measured before and intermittently after the alcohol dosage. In the top two plots, the mean PMT for each reaction time test was plotted in red over the session and the subjects’ BAC was plotted in blue. The bottom plot is for a control participant who did not consume alcohol. Baseline PMT was estimated from measurements conducted days before or days after the alcohol administration.

Among controls, PMT and RT were strongly correlated (r = 0.87, p < 0.001), shown in Fig 9, suggesting a consistent relationship between these two parameters and a consistent electromechanical delay. Changes in PMT were also assessed at an individual level over time as BAC increased. Mean PMT and BAC are plotted on separate axes as a function of time for two study participants and one control in Fig 10. Each data point in the PMT plot is the mean value across one three-minute measurement. Data for all control subjects is plotted in S1 Fig and for all study participants in S2 Fig.

The baseline PMT is the average value of all results collected during an earlier session prior to the experimental condition and is denoted as a dashed red line on the plots. Most participants’ BAC and PMT peak between 1 and 2 hours post alcohol administration and display a similar rate of rise for the BAC and PMT values as well as a similar rate of return to baseline.

**Fig 11:**
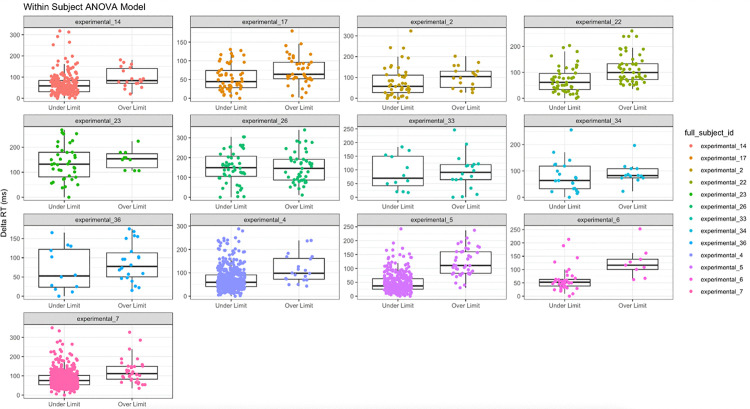
A repeated measures ANOVA showed a significant effect of BAC category on Delta RT. The “Under Limit” condition represents participants with BAC < 0.08% (not legally intoxicated), while the “Over Limit” condition represents the same participants when their BAC ≥ 0.08% (legally intoxicated). Seven of 13 participants (subjects 4, 5, 6, 7, 14, 17, and 22) demonstrated statistically significant differences in Delta RT between the two BAC conditions.

A repeated measures ANOVA was to evaluate the within-subject effect of intoxication (BAC ≥ 0.08%) on Delta RT (Fig 11). Reaction times were significantly slower when participants were intoxicated compared to when they were below the legal limit, F(1, 2161) = 156, p = 0.001, η² = 0.067. Specifically, the mean Delta RT below the legal limit was 73.21 ms (95% CI, 70.75--75.67), which increased by an estimated 39.16 ms (95% CI, 33.01–45.31) once participants crossed the legal threshold. Seven of 13 participants (subjects 4, 5, 6, 7, 14, 17, and 22) demonstrated statistically significant within-subject differences between conditions. These results indicate a robust and consistent effect of legal intoxication on the slowing reaction time.

## Discussion

The Pison wearable device detected impairment in participants through measuring increases of reaction time concomitant with increases in blood alcohol concentration. Increases in premotor reaction time that occur after a moderate dose of alcohol are representative of a slowing of cognitive processes and have been demonstrated in prior laboratory-based studies using simultaneous EMG and EEG data collection [20]. The pilot results presented in this study demonstrate a potential solution to reliably and accurately measure premotor time in real-world settings using a wearable having the footprint of a smartwatch. By monitoring reaction time unobtrusively in an easy-to-use device, individuals who are impaired by alcohol consumption can be prevented from operating a motor vehicle. Several limitations should be considered when interpreting these pilot study results. The sample size (n = 19) was small and there was an uneven distribution of participants between alcohol (n = 14) and control (n = 5) groups. Also, race or ethnicity was not collected or incorporated into analysis. These factors reduce the generalizability of results and increase the risk that individual differences may have influenced the findings. Future research should build on these pilot findings by incorporating a larger and more diverse sample to strengthen confidence in the observed effects of alcohol consumption on reaction time. Based on preliminary results, a sample of approximately 120 participants is recommended. For a two-group between-subjects design, this would mean recruiting about 60 participants per group, with oversampling to account for potential attrition. For a two-condition within-subjects design, targeting at least 100 participants to complete the study, an enrollment closer to 120 would help ensure sufficient power and data quality. A larger study will also provide opportunities to examine whether the effects vary across populations, task conditions, or contextual factors, resulting in a deeper understanding of how alcohol impacts measurable performance. Additionally, participants contributed multiple data points over time, but the analyses did not fully account for the repeated nature of the measurements or for possible confounding influences such as sex, body weight, or previous experience with the device. Future studies should use statistical models, such as mixed-effects regression, that better account for within-subject variation and potential covariates.

Another limitation of this pilot study is the choice to combine data from all participants, including those in the control group who did not consume alcohol. We did this to strengthen the statistical power given the small sample size (n = 19). Still, this decision may have added variability to the results because the two groups experienced different conditions. In an ideal setting, we would have run sensitivity analyses to test whether the findings held within each subgroup, but the small number of participants made this impractical. Future studies with larger and more evenly balanced samples will make it possible to examine subgroups separately and better assess how BAC relates to RT. Also, because no corrections beyond Tukey’s HSD were used for multiple comparisons, there is a potential risk of inflated Type I error, which should be considered when interpreting these findings.

In the analysis presented, a simple linear regression was implemented to evaluate if the measured BAC could predict the change in reaction time as measured through the Pison wearable. Although BAC was a significant predictor of the change in reaction time, (F(1, 3547) = 323.3, p < 0.001), BAC only explained 8.4% of the variance. Several factors in addition to alcohol consumption are known to impact reaction time and include age, gender, physical fitness, fatigue, presence of distractions, hydration, and modality of sensory stimulus input, among others [21,22]. Many of these factors were consistent during the experimental session, but additional observed variance may be due to a known interaction among alcohol consumption, dehydration, and fatigue through the hormone vasopressin [23]. Future studies should measure vasopressin levels prior to alcohol consumption. In addition, some participants collected baseline data in home settings, which may have differed in terms of ambient lighting or electromagnetic interference than the data collected in the laboratory setting. Future studies should have all participants collect data in real-world settings so that the impact of environmental factors as well as other factors that may influence reaction time can be assessed. This is crucial for building an ecologically valid tool for improving driving safety.

**Fig 12:**
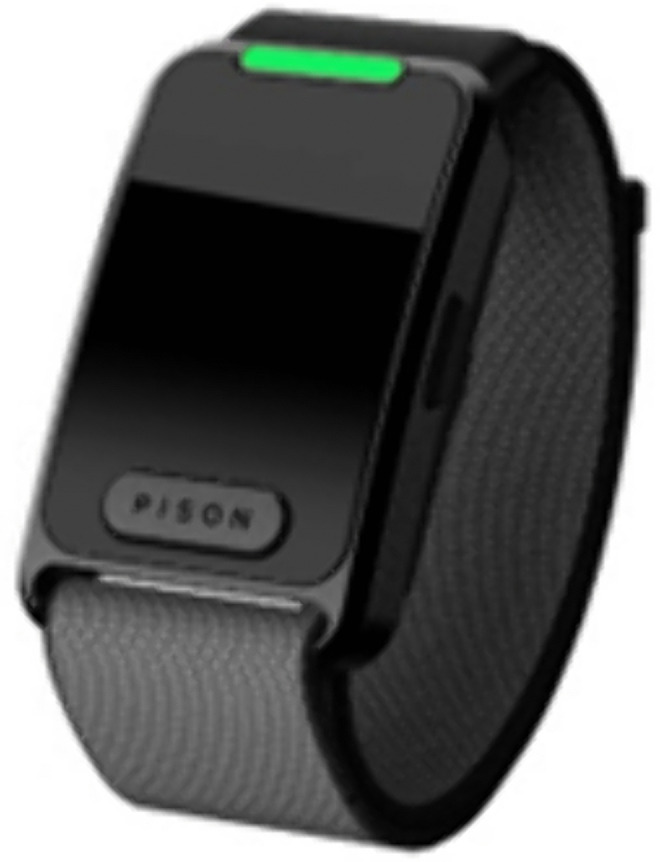
The Pison wearable sensor platform uses patented sensing technology to acquire biopotential, motion, and other physiological signals. Data from Pison’s surface electromyography, inertial measurement unit, photoplethysmography, electrocardiography, galvanic skin response, temperature, and ambient light sensors are transmitted to a client device (e.g., mobile phone) via Bluetooth Low Energy. Reprinted with permission from Pison Technology, original copyright 2024.

Pison would like to conduct additional studies using its latest device, shown in Fig 12, that includes EMG and IMU sensors implemented on a readout integrated circuit developed in partnership with STMicro. Future studies will also compare the sensitivity of EMG and IMU measures to intoxication level as well as evaluate a composite measure of PMT and RT to identify intoxication.

Overall, the neurophysiologic data measured with the Pison wearable has shown promise to determine, at a population level, in a statistically significant manner, the difference between subjects below and above the legal blood alcohol limits for operating a motor vehicle. Additionally, at an individual level, a very robust mirroring of reaction time to blood alcohol level was shown, providing value in determining an individual’s cognitive ability to engage in critical tasks, such as driving. Future adoption of this technology, or any other passive or active alcohol sensor, will depend on achieving a high degree of reliability in detecting intoxicated drivers while also avoiding false positives that would prevent non-intoxicated drivers from using their cars.

In comparing alcohol-detection methods, each technology has advantages and disadvantages. The leading candidates for ignition-interlock devices include non-dispersive infrared sensors, which are completely passive but suffer from air dilution, and near-infrared spectroscopy, whose results are complicated by melanin and hydration. Specific advantages for wearable reaction time sensors include ease of data collection and lack of calibration requirement. One disadvantage of wearable reaction time sensors is that they require active participation from users. Breathalyzers will have greater accuracy in measuring blood alcohol concentration than reaction time sensors but only have a single function and are not sensitive to impairments caused by fatigue or abuse of other substances. Reaction time sensors may be preferrable to consumers because they also function as a watch and a heart-rate and activity sensor. A combination of sensors or repeated measures from a given sensor may also be advantageous to increase performance. Emerging techniques, such as transdermal vapor sensors or camera-based systems that monitor the eyes, may be used to verify the potential intoxication suggested by a wearable or passive alcohol sensor.

Questions remain about the implementation, incentivization, enforcement, and legal liability of guidance given by consumer inebriation sensors based on reaction time. Smart cars having ignition-interlock devices that interface with a reaction-time test would be the strongest implementation. Such a model could be marketed as a new standard safety feature to help promote adoption. Self-driving cars may require reaction-time tests, or other sensor-based ignition interlock devices, before enabling a “manual” driving mode. Insurance discounts would help incentivize adoption by consumers. Bartenders could act as enforcers and use the reaction-time device as a guide for serving additional rounds to customers who may be inebriated. Discounts on liquor liability insurance to establishments that use such tests would help accelerate adoption. Taxi or ride-share services that would gain passengers may promote the use of such wearables. Alternatively or in addition, consumers themselves may be motivated to use a reaction-time sensor after drinking. In one implementation, a suggestion would be made not to drive and a family member or friend would be notified of a person’s presumed intoxication. Insurance discounts may help adoption; individuals who have been convicted of intoxicated driving previously or those aged 21 years may be required to use such a wearable device during a probation period.

## Conclusion

In this study, the first on-body, real-time measurements of impairments associated with increasing blood alcohol content were conducted using neuro-physiologic Pison sensors. This study is the first to provide insight into the influence of neural sensors and computational power to inform impairment due to alcohol. Preventing motor vehicle use while under the influence of alcohol is an impactful use case and future studies will expand the subject size as well as broaden the sources of impairment beyond alcohol to include other legal substances such as tetrahydrocannabinol, illegal substances, as well as sleep deprivation or chronic fatigue. The use of reaction time can be used to measure impairment independent of the etiology of cognitive decline.

These initial findings suggest a scalable solution to prevent driving under the influence of alcohol, exponentially reducing the risk to intoxicated drivers, passengers, other drivers, and pedestrians, thereby improving public safety. Additionally, the quantitative data produced by the Pison wearable may be used to inform casual drinkers of when they should not consume more alcohol to maintain sobriety.

## Supporting information

S1 Fig Premotor reaction time plotted as a function of time for all control subjects. Premotor time (PMT) was measured over time for five control subjects. Mean PMT for each reaction time test was plotted over the session. Baseline PMT was calculated from measurements performed days before or days after the in-person session.
(PDF)

S2 Fig Blood alcohol concentration and premotor reaction time plotted as a function of time. Blood alcohol and premotor time (PMT) were measured before and intermittently after the alcohol dosage. Plots are shown for all 14 participants. The mean PMT for each reaction time test was plotted over the session as well as the subject’s BAC. Baseline PMT was calculated from measurements performed days before or days after the in-person session.
(PDF)
