# Supplementary figures and images for: On-body measure of reaction time correlates with intoxication level
Source: PLoS One. 2026 Apr 15;21(4):e0323858. doi: 10.1371/journal.pone.0323858 (PMC13082662; doi:10.1371/journal.pone.0323858)

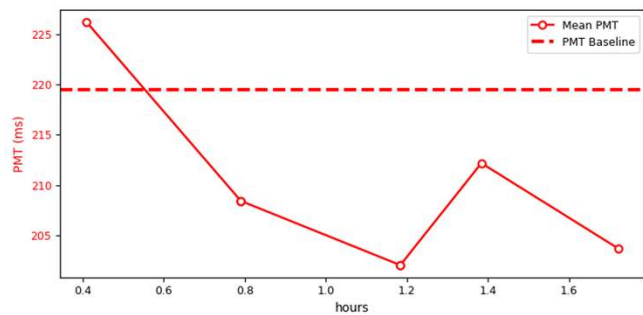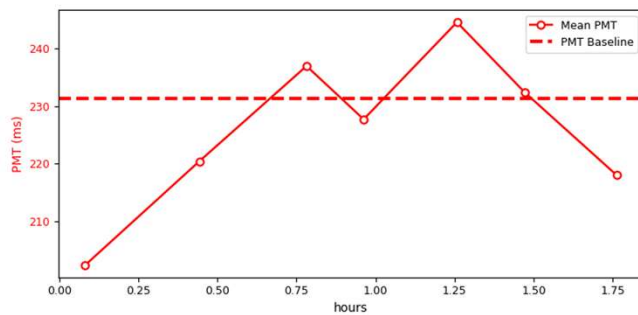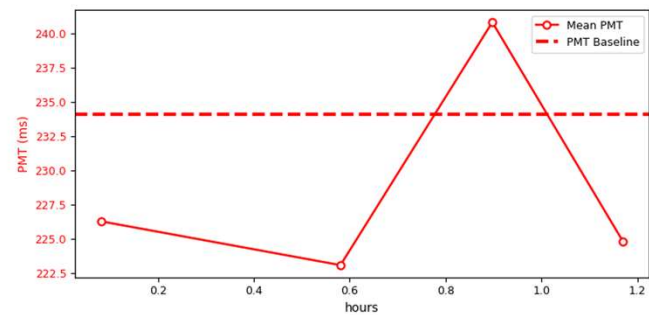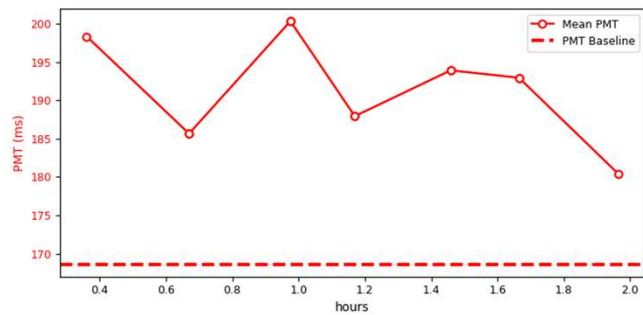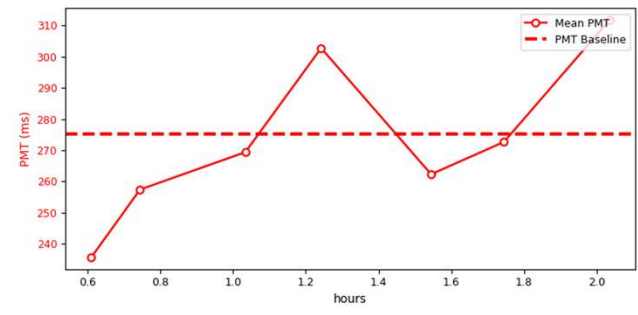

Supplement: S1 Fig — Premotor time (PMT) was measured over time for five control subjects. Mean PMT for each reaction time test was plotted over the session. Baseline PMT was calculated from measurements performed days before or days after the in-person session. (PDF) [file pone.0323858.s001.pdf]
