# Supplementary material for: On-body measure of reaction time correlates with intoxication level
Source: PLoS One. 2026 Apr 15;21(4):e0323858. doi: 10.1371/journal.pone.0323858 (PMC13082662; doi:10.1371/journal.pone.0323858)
Supplement: S2 Fig — Blood alcohol and premotor time (PMT) were measured before and intermittently after the alcohol dosage. Plots are shown for all 14 participants. The mean PMT for each reaction time test was plotted over the session as well as the subject’s BAC. Baseline PMT was calculated from measurements performed days before or days after the in-person session. (PDF) [file pone.0323858.s002.pdf]

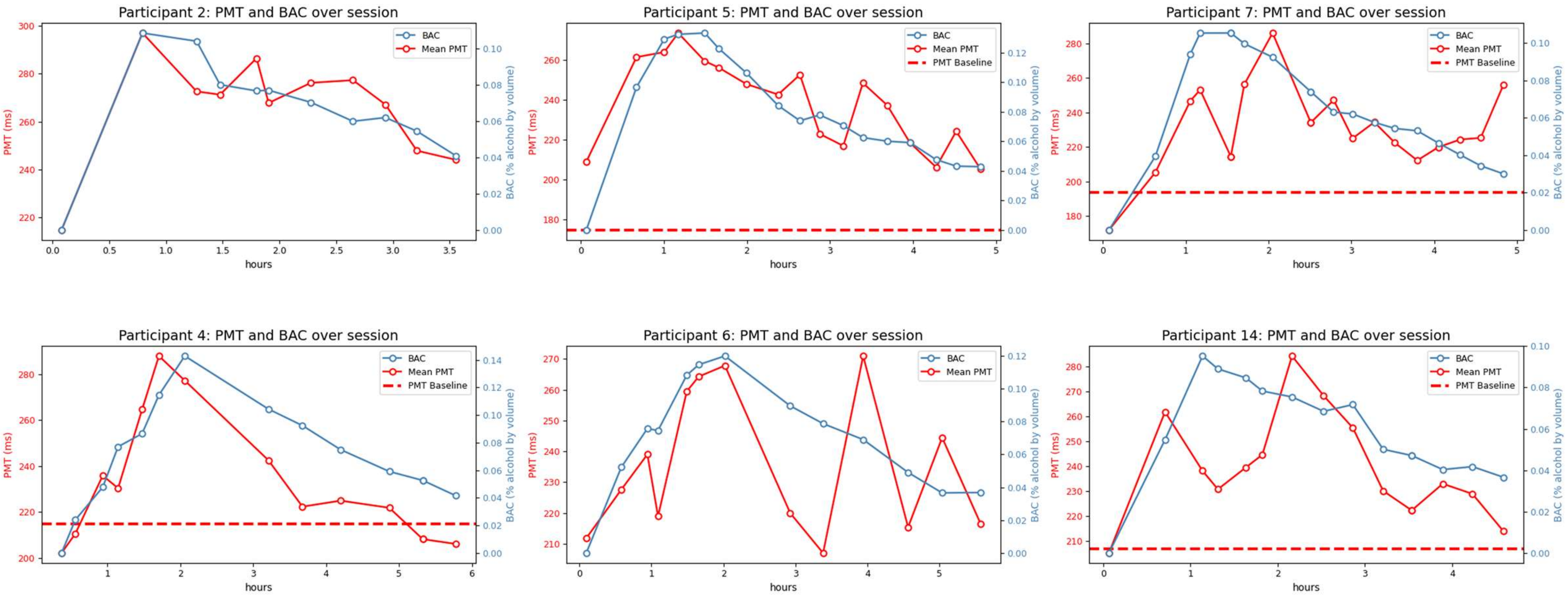

Participant 2: PMT and BAC over session
Participant 5: PMT and BAC over session
Participant 7: PMT and BAC over session
Participant 4: PMT and BAC over session
Participant 6: PMT and BAC over session
Participant 14: PMT and BAC over session
BAC
Mean PMT
PMT Baseline
PMT (ms)
BAC (% alcohol by volume)
hours

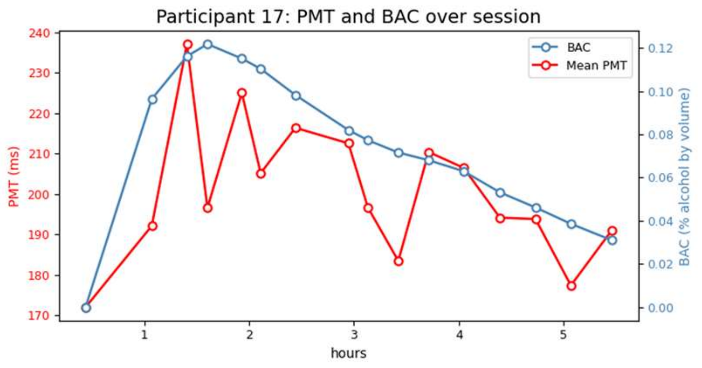
Participant 17: PMT and BAC over session
BAC
Mean PMT
PMT (ms)
BAC (% alcohol by volume)
hours

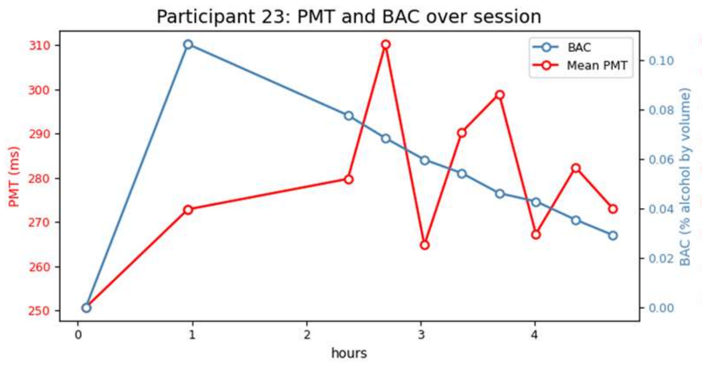
Participant 23: PMT and BAC over session
BAC
Mean PMT
PMT (ms)
BAC (% alcohol by volume)
hours

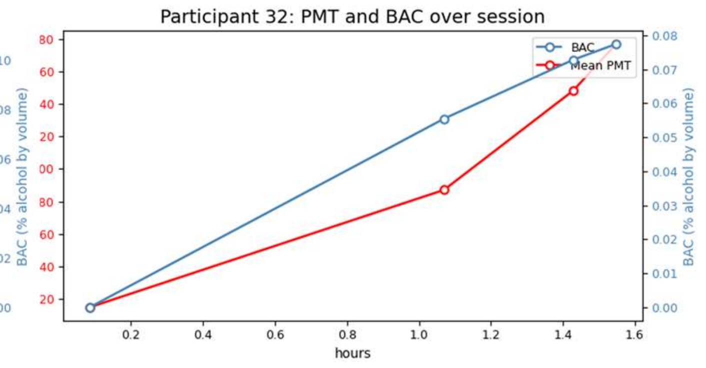
Participant 32: PMT and BAC over session
BAC
Mean PMT
PMT (ms)
BAC (% alcohol by volume)
hours

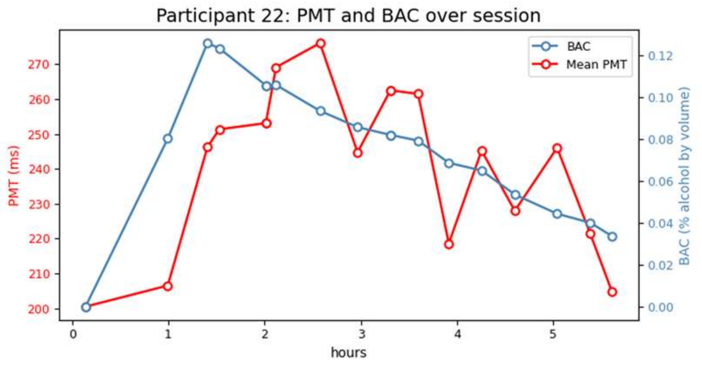
Participant 22: PMT and BAC over session
BAC
Mean PMT
PMT (ms)
BAC (% alcohol by volume)
hours

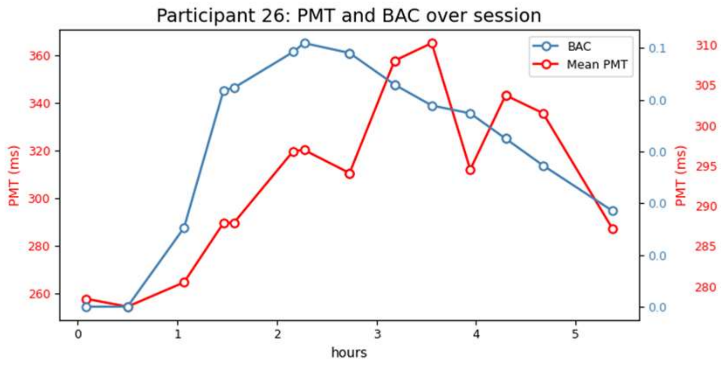
Participant 26: PMT and BAC over session
BAC
Mean PMT
PMT (ms)
BAC (% alcohol by volume)
hours

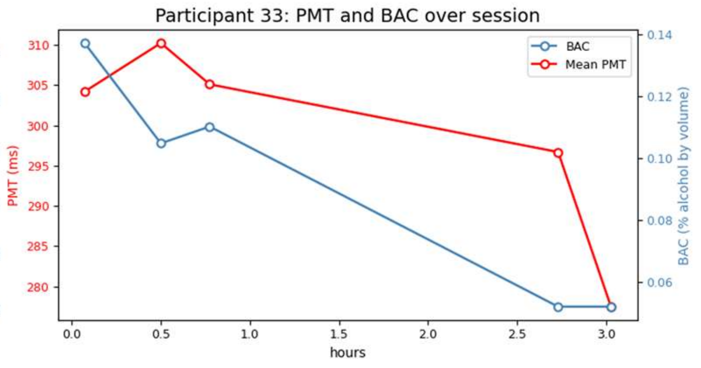
Participant 33: PMT and BAC over session
BAC
Mean PMT
PMT (ms)
BAC (% alcohol by volume)
hours

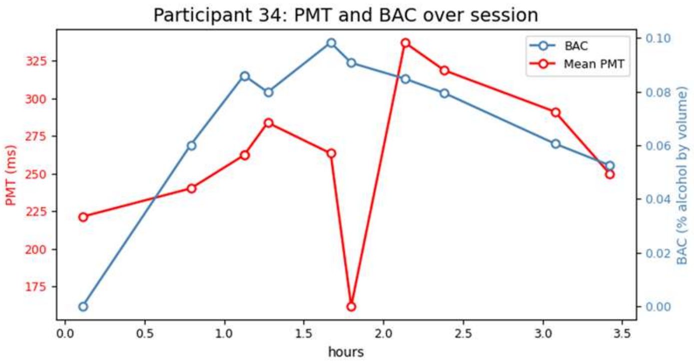

Participant 34: PMT and BAC over session
BAC
Mean PMT
PMT (ms)
BAC (% alcohol by volume)
hours
175
200
225
250
275
300
325
0.00
0.02
0.04
0.06
0.08
0.10
0.0
0.5
1.0
1.5
2.0
2.5
3.0
3.5

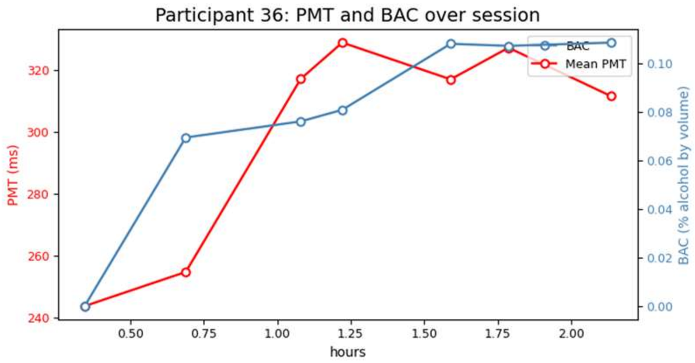

Participant 36: PMT and BAC over session
BAC
Mean PMT
PMT (ms)
BAC (% alcohol by volume)
hours
240
260
280
300
320
0.00
0.02
0.04
0.06
0.08
0.10
0.50
0.75
1.00
1.25
1.50
1.75
2.00
